# ﻿Three new ramarioid species of *Phaeoclavulina* (Gomphaceae, Gomphales) from China

**DOI:** 10.3897/mycokeys.108.128716

**Published:** 2024-08-21

**Authors:** Peng-Tao Deng, Wen-Hao Liu, Zai-Wei Ge, Ping Zhang

**Affiliations:** 1 College of Life Science, Hunan Normal University, Changsha 410081, China Hunan Normal University Changsha China; 2 CAS Key Laboratory for Plant Diversity and Biogeography of East Asia, Kunming Institute of Botany, Chinese Academy of Sciences, Kunming, Yunnan, 650201, China Kunming Institute of Botany, Chinese Academy of Sciences Kunming China

**Keywords:** Morphological characters, phylogenetic analysis, Ramarioid fungi, taxonomy

## Abstract

Three new species of *Phaeoclavulina* from China are described: *Phaeoclavulinabicolor*, *P.echinoflava*, and *P.jilinensis*. Recognition of the new species is supported by morphological and molecular evidence. Phylogenetic analyses of concatenated ITS1–5.8S–ITS2 and nuclear large subunit sequences support the establishment of the new species and their placement within the *Phaeoclavulina* clade. A key to the known *Phaeoclavulina* species in China is provided.

## ﻿Introduction

Brinkmann (1897) first proposed the genus *Phaeoclavulina* Brinkmann to accommodate a coral fungus with spiny spores, and described *Phaeoclavulinamacrospora* Brinkmann, as the type species, characterized by a branched fruiting body, ochraceous spores, and 2-spored basidia. The genus has since been expanded to include certain gomphoid fungi formerly classified in *Gomphus* Pers. and ramarioid fungi previously placed in Ramariasubg.Echinoramaria Corner. Thus, the morphology of the *Phaeoclavulina* fruiting body may be ramaroid, unipileate, or merismatoid (Giachini and Castellano 2011). Although *Phaeoclavulina* exhibits diversity in the morphology of the fruiting body, the microstructure is more homogeneous: monomitic hyphae, clamp connections in hyphae and basidia, and echinulate (mostly with acute spines) to verrucose, subreticulate or reticulate spores (Giachini 2004; Giachini and Castellano 2011). In the past decade, numerous new species of *Phaeoclavulina* have been described: 12 species were described by Franchi and Marchetti from Italy, and three species were described from China and Mexico (Franchi and Marchetti 2018, 2019, 2020; González-Vila et al. 2020; Liu et al. 2022).

Species of *Phaeoclavulina* have been described from all over the world, but they are mainly distributed in tropical, subtropical, and temperate regions, and grow in coniferous, broad-leaved, or mixed forest with basidiomata occurring in summer and autumn. Currently, no evidence shows that *Phaeoclavulina* species are mycorrhizal fungi, but their growth on decaying wood indicates that the species may be saprophytic. A number of ramarioid fungi in *Phaeoclavulina* are edible (Li JZ 2008; Dai YC et al. 2010), and have a delicious taste and relatively large fruiting body. Examples of edible taxa are *P.abietina* (Pers.) Giachini, *P.longicaulis* (Peck) Giachini, *P.cyanocephala* (Berk. & M.A. Curtis) Giachini, and *P.campestris* (K. Yokoy. & Sagara) Giachini.

In China, previous studies have reported the occurrence of 18 species of *Phaeoclavulina*: *P.abietina*, *P.aeruginea* P. Zhang, *P.capucina* (Pat.) Giachini, *P.campestris*, *P.cinnamomea* W.Q. Qin, *P.cokeri* (R.H. Petersen) Giachini, *P.curta* (Fr.) Giachini, *P.cyanocephala*, *P.decolor* (Berk. & M.A. Curtis) Giachini, *P.eumorpha* (P. Karst.) Giachini, *P.flaccida* (Fr.) Giachini, *P.grandis* (Corner) Giachini, *P.longicaulis*, *P.macrospora*, *P.mutabilis* (Schild & R.H. Petersen) Giachini, *P.sikkimia* (S.S. Rattan & Khurana) Giachini, *P.viridis* (Pat.) Giachini, and *P.zippelii* (Lév.) Overeem (Teng 1963; Li JZ 2008; Liu et al. 2022). Given that *Phaeoclavulina* was formerly included in *Ramaria*, most records of *Phaeoclavulina* species in China are based on previous studies of *Ramaria*. There is a lack of systematic research on *Phaeoclavulina* in China, from where only three new species have been discovered: *Ramarialuteoaeruginea* P. Zhang & Zhu L. Yang (assignable to *Phaeoclavulina* as that genus is currently circumscribed), *P.cinnamomea*, and *P.aeruginea* (Zhang et al. 2005; Liu et al. 2022). Therefore, a comprehensive survey combined with morphological and molecular research is needed to conduct a systematic analysis of *Phaeoclavulina* and to understand the *Phaeoclavulina* species diversity in China.

During research on ramarioid and coralloid fungi in China, seven specimens of *Phaeoclavulina* were collected. On the basis of their morphology and molecular phylogenetic analysis, these specimens were identified as three new species of *Phaeoclavulina*, which are formally described herein as *P.bicolor*, *P.echinoflava*, and *P.jilinensis*.

## ﻿Materials and methods

### ﻿Specimen sources

Seven specimens of *Phaeoclavulina* were gathered by the authors from 2004 to 2021 in Xizang Autonomous Region, Jilin Province, and Hainan Province. The fresh fruiting body characters and habitat were recorded in the field, including whether the color changed when injured. The fresh basidiomata were dried at 55–60 °C or desiccated in silica gel. The dried samples are deposited in the Mycological Herbarium of Hunan Normal University (**MHHNU**), Changsha, China, and the Herbarium of Kunming Institute of Botany, Chinese Academy of Sciences (**KUN-HKAS**), Kunming, China.

### ﻿Morphological observation

Macroscopic features of the newly collected specimens were described from the fresh fruiting body, record sheet, and photographs. The colors reported in the descriptions were determined following Kornerup and Wanscher (1978) and Ridgway (1912). Dried fruiting body sections were placed in 5% KOH solution, containing 1% Congo red solution and cotton blue, to observe the ornamentation of the spores. Microscopic characters were observed from a portion of a dried fruiting body with a light microscope, including spores, basidia, and hyphae. In the description of basidiospores, 60 basidiospores were measured for each species, and their size is expressed in the form (a–) b–c (–d), where ‘a’ and ‘d’ are the minimum and maximum dimensions of spores, respectively, and ‘b’ and ‘c’ are the range representing the majority of the spore dimensions. The abbreviation [n/m/p] indicates that the measurements were obtained from ‘n’ basidiospores from ‘m’ basidiomata of ‘p’ specimens. The Q value represents the length-to-width ratio of basidiospores, and the Q_m_ value is the average Q ± standard deviation.

### ﻿DNA extraction, PCR amplification, and sequencing

Genomic DNA was extracted from dried specimens using the EZup Column Fungal Genomic DNA Extraction Kit (Sangon Biotech, Shanghai, China). A sample (25–30 mg) of a dried specimen was ground to powder in liquid nitrogen in accordance with the manufacturer’s instructions. The primer pairs ITS4/ITS5 and LR5/LR0R were used to amplify the nuclear rDNA ITS1–5.8S–ITS2 (ITS) and nuclear large subunit (LSU) regions, respectively (Vilgalys and Hester 1990; White et al. 1990; Gardes and Bruns 1993). The PCR amplification reactions were performed on an Eppendorf Mastercycler thermal cycler in a 25 µL volume containing 1 µL DNA, 2 µL primers, 9.5 µL ddH_2_O, and 12.5 µL 2× Es Taq Master Mix. The amplification procedure consisted of pre-denaturation at 94 °C for 4 min, then 32 cycles comprising denaturation at 94 °C for 40 s, annealing at 55 °C (ITS) or 52 °C (LSU) for 40 s, and extension at 72 °C for 1 min, followed by a final extension at 72 °C for 8 min, and storage at 4 °C (Liu et al. 2022). An ABI 3730 DNA Analyzer (PerkinElmer Inc., USA) was used to sequence the PCR products. All PCR products were separated by electrophoresis in 1% agarose gel and then submitted to Sangon Biotech (Shanghai, China) for sequencing. The newly generated sequences (seven ITS and seven LSU) were deposited in GenBank (accession numbers are listed in Table 1).

**Table 1.** Details of the ITS and 28S rDNA sequences used for phylogenetic analyses. The sequences newly generated in this study are highlighted in bold.

Taxon	Voucher	GenBank No. ITS	GenBank No. LSU	Geographical origin	References
*Phaeoclavulinaabietina*	OSC 134649	JX310378	JX287478	USA	Unpublished
*P.abietina*	OSC 140661	JX310379	JX287479	USA	Unpublished
*P.aeruginea*	MHHNU8909	ON262784	ON262781	China	Liu et al. (2022)
*P.aeruginea*	MHHNU6887	ON262785	ON262782	China	Liu et al. (2022)
*P.alboapiculata*	AMB 18590	MT055971	MT053248	Italy	Unpublished
*P.alboapiculata*	AMB 18585	MT055964	–	Italy	Unpublished
*P.alboapiculata*	AMB 18613	MT452509	–	Italy	Unpublished
***P.bicolor***	**MHHNU10702**	**PP809798**	**PP800475**	**China**	**This study**
***P.bicolor***	**MHHNU10703**	**PP809799**	**PP800476**	**China**	**This study**
*P.cinnamomea*	MHHNU10376	ON262786	ON262783	China	Liu et al. (2022)
*P.cyanocephala*	TH9064	KT339249	KT339290	Guyana	Unpublished
*P.coniferarum*	AMB 18562	MT055942	–	Italy	Unpublished
*P.coniferarum*	AMB 18531	NR_176718	NG_088119	Italy	Unpublished
*P.carovinacea*	AMB 18533	NR_176719	–	Italy	Unpublished
*P.carovinacea*	AMB 18551	MT055933	–	Italy	Unpublished
*P.carovinacea*	AMB 18534	MT055918	–	Italy	Unpublished
*P.carovinacea*	TUR-A 209584	ON561378	ON530902	Italy	Unpublished
*P.clavarioides*	PRM:945441	LR723647	–	Czech	Kříž et al. (2019)
*P.clavarioides*	PRM:945440	LR723646	LR723645	Czech	Kříž et al. (2019)
*P.curta*	AMB 18641	MW115423	MW092704	Italy	Unpublished
*P.curta*	AMB 18605	MT452501	–	Italy	Unpublished
*P.curta*	UBC F32034	KX236126	–	Canada	Unpublished
*P.curta*	HAY-F-000746	PP294846	–	USA	Unpublished
***P.echinoflava***	**HKAS 45984**	**PP809801**	**PP800478**	**China**	**This study**
***P.echinoflava***	**HKAS 45992**	**PP809800**	**PP800477**	**China**	**This study**
*P.flaccida*	AMB n. 18209	MF288928	MF288936	Italy	Unpublished
*P.gigantea*	FH109	–	AY574703	USA	Giachini et al. (2010)
*P.insignis*	FH104	–	AY574704	USA	Giachini et al. (2010)
***P.jilinensis***	**MHHNU9149**	**PP809802**	**PP800479**	**China**	**This study**
***P.jilinensis***	**MHHNU9164**	**PP809803**	**PP800480**	**China**	**This study**
***P.jilinensis***	**MHHNU10504**	**PP809804**	**PP800481**	**China**	**This study**
*P.minutispora*	AMB 18588	MT055969	MT053246	Italy	Unpublished
*P.minutispora*	AMB 18586	MT055965	MT053243	Italy	Unpublished
*P.macrospora*	AMB 18614	MT452510	–	Italy	Unpublished
*P.pseudozippelii*	BBH 43575	MG214661	MG214663	Thailand	Wannathes et al. (2018)
*P.roellinii*	PRM:945445	LR723649	–	Czech	Kříž et al. (2019)
*Ramariaadmiratia*	TENN 69114	NR_137862	NG_059504	USA	Hughes et al. (2014)
*R.calvodistalis*	TENN 69095	KJ416132	KJ416135	USA	Hughes et al. (2014)

### ﻿Alignment and phylogenetic analysis

Full sequences of the two DNA regions (ITS and LSU) obtained from the seven samples in this study, together with sequences for 31 accessions publicly accessible in GenBank, were used to construct a multigene dataset. *Ramariaadmiratia* R.H. Petersen and *Ramariacalvodistalis* R.H. Petersen were selected as the outgroup owing to their phylogenetic placement external to the *Phaeoclavulina* clade (Xu et al. 2022). The final ITS+LSU dataset comprised 62 sequences (36 ITS and 26 LSU; Table 1) and was used for a multigene phylogenetic analysis. The sequences were aligned using MAFFT 7.471 with the default settings for gap insertion and extension penalties (Katoh and Standley 2016), then manually modified where necessary with BIOEDIT v7.2.5 (Hall 1999). The final concatenated ITS+LSU dataset was generated by SEQUENCEMATRIX v1.7.8 (Vaidya et al. 2011). Maximum likelihood (ML) analysis of the dataset was conducted with RAxML v7.2.6 (Stamatakis et al. 2005; Stamatakis 2006), using a GTR+G evolutionary model (Stamatakis et al. 2008). A bootstrap (BS) analysis was performed with 1000 replicates to assess support for the ML tree topology. Bayesian inference (BI) was performed using MRBAYES v3.2.7 (Ronquist et al. 2012). The optimal evolutionary model (GTR+I+G) was selected based on the Akaike information criterion with MRMODELTEST v2.4 (Nylander 2004). Four independent Markov chain Monte Carlo (MCMC) chains were run, with sampling every 100 generations, for a total of three million generations (standard deviation ≤ 0.01). Posterior probabilities (PP) were calculated after discarding the first 25% of generations as burn-in. FIGTREE v1.4.2 (Rambaut 2012), PHOTOSHOP CS6, and ILLUSTRATOR CS5 (Adobe Systems, Inc., San Jose, CA, USA) were used to visualize and adjust the phylogenetic trees.

## ﻿Results

### ﻿Phylogenetic analysis

**Figure 1.**
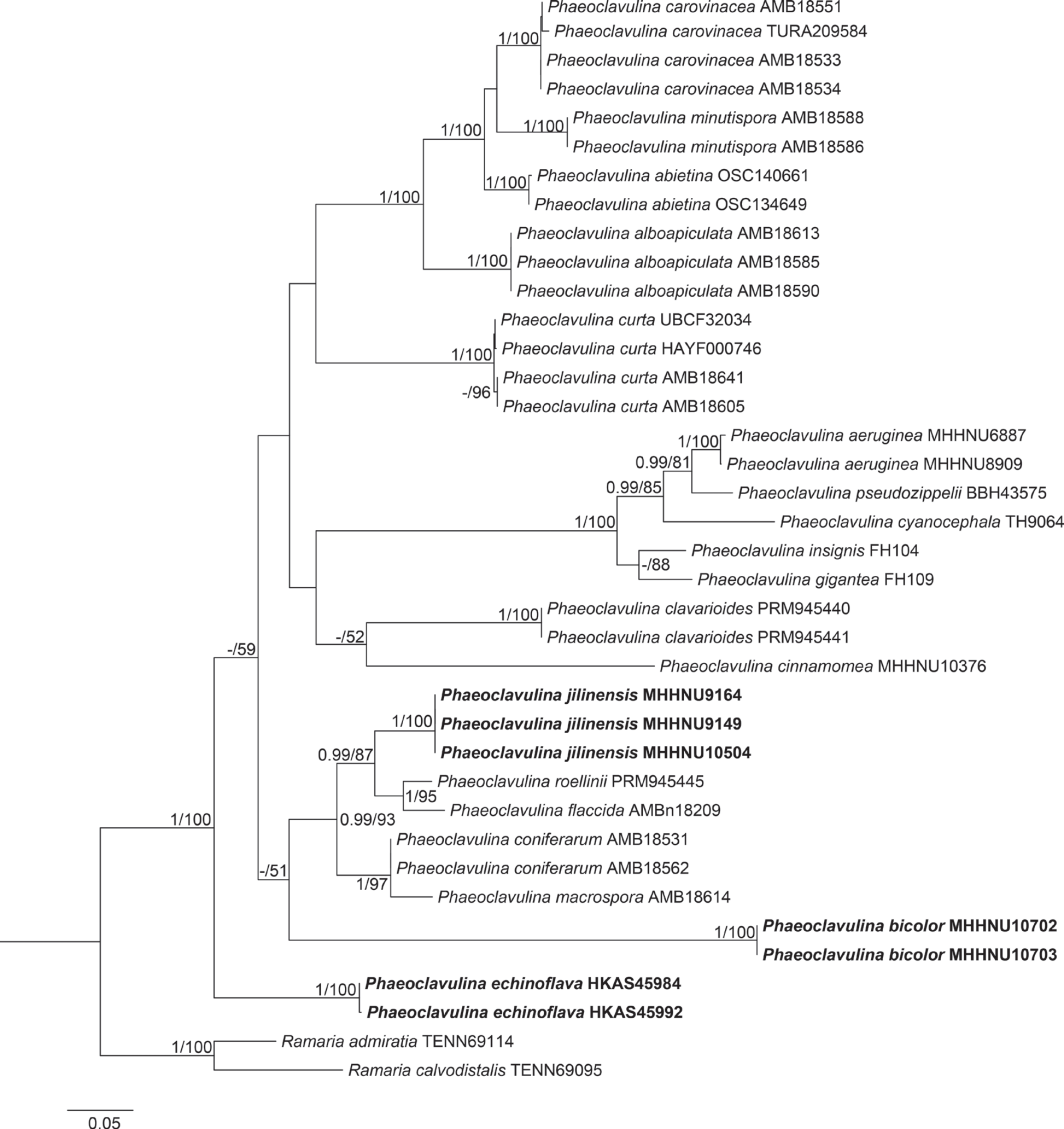
Phylogenetic relationships of *Phaeoclavulina* species inferred from a concatenated ITS and LSU sequence dataset under the maximum likelihood optimality criterion. Bayesian posterior probabilities (PP) > 0.90 and bootstrap values (BS) > 50% are reported at the nodes (PP/BS); “–” indicates that the support value was less than the respective threshold. The three new species from China are highlighted in bold.

The final ITS+LSU dataset comprised 1661 aligned positions in total. The BI phylogeny (not shown) was extremely similar in topology and branch support to the ML tree. The ML phylogeny constructed from the ITS+LSU dataset (Fig. 1) revealed that 38 samples were placed in a strongly supported (PP 1, BS 100%) *Phaeoclavulina* clade. The seven samples newly sequenced in this study were placed on three distinct branches, each with strong statistical support (PP 1, BS 100%). *Phaeoclavulinajilinensis*, *P.flaccida*, and *P.roellinii* (Schild) Giachini were grouped in a well-supported subclade (PP 0.99, BS 87%). *Phaeoclavulinabicolor* was placed as sister to a clade comprising the three preceding species together with *P.macrospora* and *P.coniferarum* Franchi & M. Marchetti but with no support. The sequences of *P.echinoflava* were placed in an isolated position sister to all other samples within the *Phaeoclavulina* clade. On the basis of these results, three new species of *Phaeoclavulina* based on the newly collected Chinese specimens are proposed.

### ﻿Taxonomy

#### ﻿ Phaeoclavulina bicolor

Taxon classificationFungiGomphalesGomphaceae

P. Zhang & W.H. Liu sp. nov.

A709E437-F933-5761-B6B7-54C3EA15975E

854010

Fig. 2

##### Diagnosis.

Differs from other *Phaeoclavulina* species by the yellowish white apex.

##### Type.

China • Hainan Province, Jianfengling National Forest Park, 18°74'21"N, 108°84'81"E, 986 m asl., 30 July 2021, leg. P. Zhang (holotype MHHNU10702).

##### Etymology.

*bicolor* (Latin), referring to the different color of the branches and branch tips.

##### Basidiomata.

Coralloid, 60–90 mm tall, 30–45 mm broad, light grayish brown when young [6D3–4], dark brown in age [6E5–7]. Stipe single, 10–20 mm long, white mycelia at the base. Branches sparse, branching from the base, dichotomous to polychotomous, divided two to three times, internodes becoming gradually shorter, terminal branches short and not flat, in cross-section rounded, axils V-shaped. Tips short and blunt, yellowish white [2A2–4] or pale white [1A1–2]. Context white, fleshy. Taste and odor, and macrochemical reactions not recorded.

**Figure 2.**
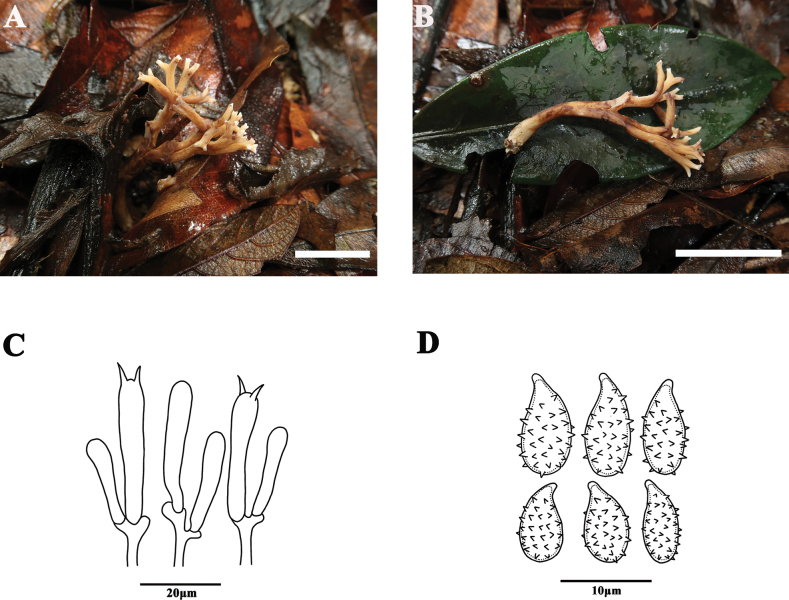
Basidiomata and microscopic features of *Phaeoclavulinabicolor* (MHHNU10702) **A, B** basidiomata **C** basidia **D** basidiospores. Scale bars: 5 cm (**A, B**).

##### Micromorphology.

Context hyphae in parallel arrangement, 3–4 µm wide, cylindric, inflated, with clamp connections but not at every septum, thin-walled, smooth, hyaline. Basidia approximately 40–50 × 5–8 µm with two sterigmata 5–6 µm long, hyaline, clavate, with clamp connection at base. Cystidia absent. Basidiospores [60/3/3] (7.6)8–10(10.5) × (3.8)4–6(6.5) µm [Q = 1.70–2.20, Q_m_ = 1.97 ± 0.18], long-ellipsoid or cylindrical, slightly thick-walled, pale yellow in KOH, cyanophilic, surface coarse, echinulate, spines 0.6–1.0 μm long, acute; hilar appendage acuminate.

##### Additional materials examined.

• Hainan Province, Jianfengling National Forest Park, 18°74'32"N, 108°84'76"E, 978 m asl., 30 July 2021, MHHNU10703.

##### Habit and distribution.

Solitary, growing on the soil of broad-leaved forest in tropical rain forest; basidiomata occur in summer. Known only from the type locality in China.

##### Comments.

*Phaeoclavulinabicolor* is distinguished from other species of *Phaeoclavulina* by the yellowish white branch tips, and the mainly grayish brown to dark brown basidiomata. *Phaeoclavulinasubdecurrens* (Coker) Franchi & M. Marchetti also has basidiomata with a different color at the tips, but in *P.subdecurrens* the branch tips are pale violet to off white. *Phaeoclavulinaaeruginea* has unique copper-green branch tips and has relatively larger spores than *P.bicolor* (13–16 × 8–9 μm vs. 8–10 × 4–6 μm). In the field, *P.cyanocephala* has relatively larger basidiomata (8–18 × 2–7 cm) and is distributed worldwide, whereas in *P.bicolor* the basidiomata is 6–9 cm tall, 3–4.5 cm broad, and the species is presently known only from Hainan Province in China.

#### ﻿ Phaeoclavulina echinoflava

Taxon classificationFungiGomphalesGomphaceae

P. Zhang & W.H. Liu sp. nov.

7B14224B-59D4-5647-8404-A50594F369D7

854011

Fig. 3

##### Diagnosis.

Differs from other *Phaeoclavulina* species by the bright yellow basidiomata when young, brownish yellow with age.

##### Type.

China • Xizang Autonomous Region, Jiangda County, 31°49'39"N, 98°21'88"E, 3600 m asl., 29 July 2004, Z.W. Ge 204 (HKAS 45984, holotype).

##### Etymology.

*echinoflava* (Latin), referring to the bright yellow fruiting body and echinulate spores.

##### Basidiomata.

Coralloid, 65–75 mm tall, 35–45 mm broad, bright yellow when young [1A6–7], yellow to pale brown with age [1A3–1B3]. Stipe single or falsely fasciculate, 20–30 mm long, yellowish white [1A2], smooth, densely white mycelia at the base. Many branches diverge from the stalk, dichotomous to polychotomous, divided three to four times, branches thick and sparse, terminal branches short, in cross-section rounded, bifurcation narrowly V- or U-shaped. Tips of branches concolorous, broom-form or short-digitate by maturity, short and blunt. Context whitish, fleshy. Taste and odor, and macrochemical reactions not recorded.

**Figure 3.**
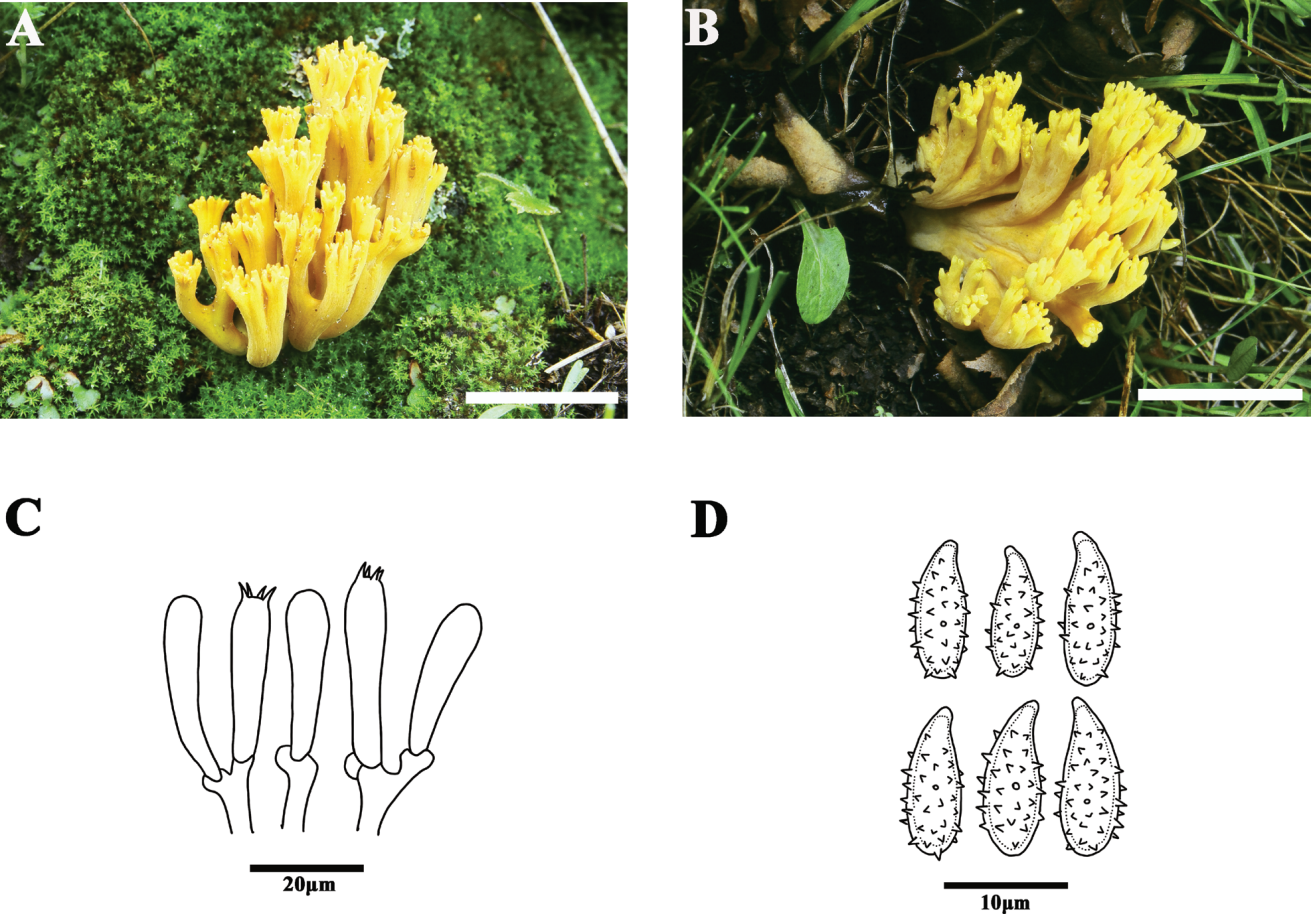
Basidiomata and microscopic features of *Phaeoclavulinaechinoflava* (HKAS 45984) **A, B** basidiomata **C** basidia **D** basidiospores. Scale bars: 5 cm (**A, B**).

##### Micromorphology.

Context hyphae in parallel arrangement, 5–8 μm wide, cylindric, inflated to 12 μm wide, clamp connections present, ampulliform clamps occasional, thin-walled, smooth, hyaline. Basidia approximately 22–34 × 6–8 µm with four sterigmata 3–5 µm long, hyaline, clavate or subclavate, with clamp connection at base. Cystidia absent. Basidiospores [60/2/2] (8.0)9.0–10.5(11.0) × (3.2)3.5–4.0(4.5) μm [Q=2.39–2.86, Qm=2.61 ± 0.21] elongate obovoid, slightly thick-walled, ochraceous in KOH, cyanophilic, surface coarse, strongly cyanophilius echinulate, spines 0.8–1.5 μm long, acute; hilar appendage inconspicuous and acuminate.

##### Additional materials examined.

• Xizang Autonomous Region, Jiangda County, 3600 m asl., 29 July 2004, Z.W. Ge 212 (HKAS 45992).

##### Habit and distribution.

Solitary, growing on the humus layer under shrubland at high altitudes; basidiomata generally occur in summer. Known only from the type locality in China.

##### Comments.

The bright yellow fruiting body of *P.echinoflava* is a distinctive character in *Phaeoclavulina*. In *Phaeoclavulina*, *P.echinovirens* (Corner, K.S. Thind & Dev) Giachini, *P.flaccida* and *P.decurrens* (Fr.) Giachini also possess a yellow fruiting body. However, in *P.flaccida* the fruiting body is beige and the basidia are relatively larger than those of *P.echinoflava* (38–45 × 5.5–6.5 μm vs. 22–34 × 6–8 μm). *Phaeoclavulinadecurrens* has a ramaroid fruiting body, ochre to buff in color fading to a white stem, and has relatively shorter basidiospores than those of *P.echinoflava* ((4.2)4.9(5.5) × (2.5)2.9(3.2) μm vs. (8.0)9.0–10.5(11.0) × (3.2)3.5–4.0(4.5) μm). *P.echinovirens* differs from *P.echinoflava* in having larger basidiomata (7.5–13 × 4.5–7.5 cm) and orange yellow tips. The phylogenetic reconstructions placed *P.echinoflava* in an isolated position within the *Phaeoclavulina* clade, which is consistent with the unique fruiting body color of *P.echinoflava*.

#### ﻿ Phaeoclavulina jilinensis

Taxon classificationFungiGomphalesGomphaceae

P. Zhang & W.H. Liu sp. nov.

0478490F-3C29-5137-AA20-E851E9F45A14

854012

Fig. 4

##### Diagnosis.

Differs from other *Phaeoclavulina* species by the citrine fruiting body and only known from Jilin Province, China.

##### Type.

China • Jilin Province, Fusong County, 42°57'66"N, 127°97'68"E, 665 m asl., 26 August 2020, leg. P. Zhang (holotype MHHNU 10504).

##### Etymology.

*jilinensis* (Latin), referring to the currently known distribution of the species in Jilin Province, China.

##### Basidiomata.

Ramaroid, 35–90 mm tall, 15–50 mm broad, pinecone yellow [2A3–5], pale lemon-yellow [4A5–6] with age. Stipe single or falsely fasciculate, 25–35 mm long, concolorous with branches when young, ochraceous with age, smooth, many mycelia at base, not changing color on bruising. Branches dichotomous to polychotomous, divided three to five times, primary branches thick and in cross-section rounded; terminal branches short and becoming flat, bifurcation narrowly V-shaped. Apices acute, rather short, dichotomous, concolorous with branches. Context whitish, fleshy. Taste and odor, and macrochemical reactions not recorded.

**Figure 4.**
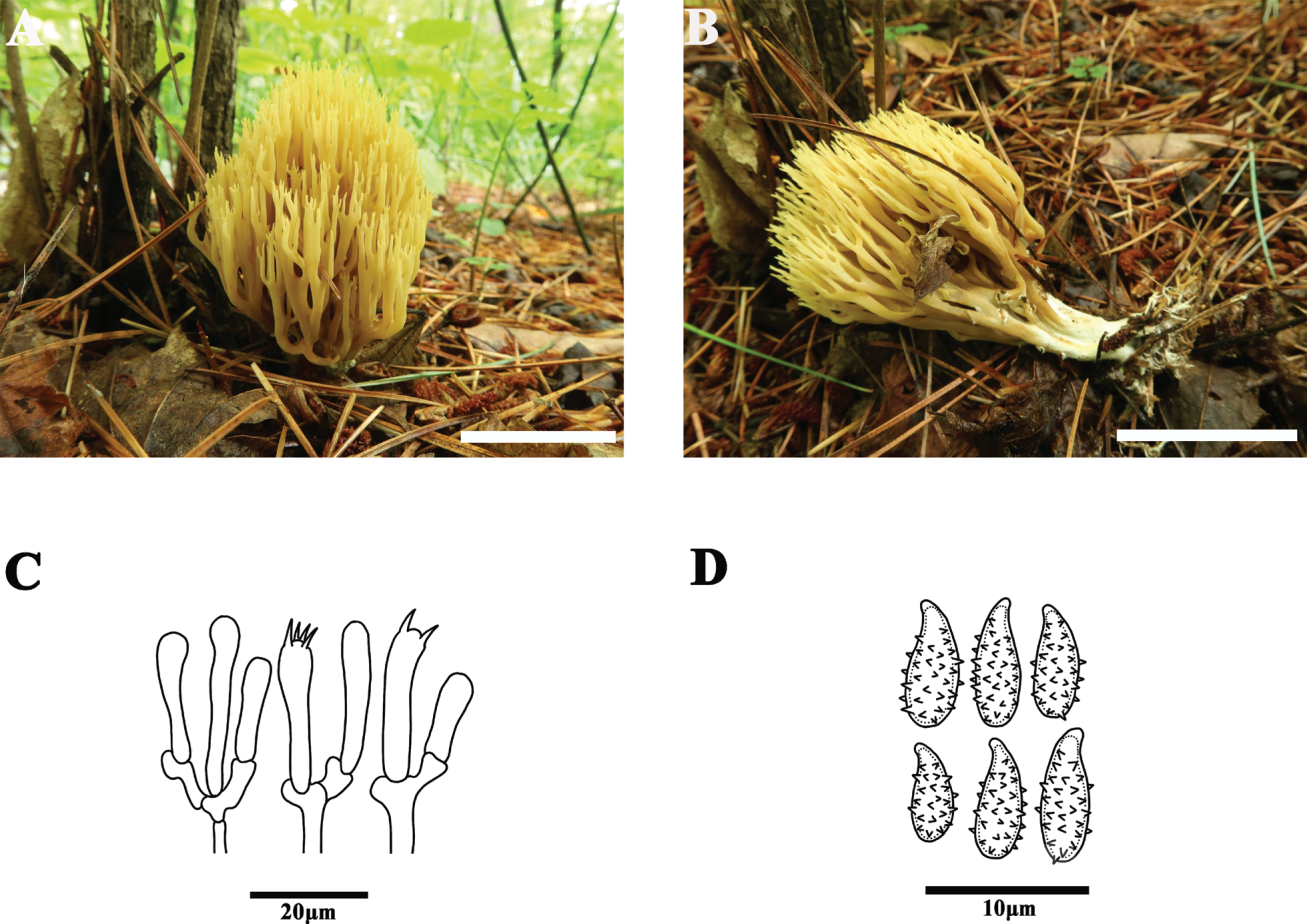
Basidiomata microscopic features of *Phaeoclavulinajilinensis* (MHHNU 10504) **A, B** basidiomata **C** basidia **D** basidiospores. Scale bars: 5 cm (**A, B**).

##### Micromorphology.

Context hyphae compact, 3–7 μm wide, cylindric, inflated to 11 μm wide, clamp connections present, thin-walled, smooth, hyaline. Basidia approximately 20–36 × 5–7 µm with four sterigmata 3–5 µm long, hyaline, clavate or subclavate, with clamp connection at base. Cystidia absent. Basidiospores [60/3/3] (5.5)6.0–8.0(8.5) × (2.8)3.0–5.0(5.5) μm [Q = 1.67–2.40, Q_m_ = 1.96 ± 0.16], elongate-ellipsoid, thick-walled, pale yellow in KOH, cyanophilic, surface coarse, with short but distinct spines, spines 0.8–1.5 μm long, strongly cyanophilous, hilar appendage distinct.

##### Habit and distribution.

Solitary on the humus layer under mixed forest; basidiomata generally occur in summer. Known only from the type locality in China.

##### Additional materials examined.

• Jilin Province, Yanbian Korean Autonomous Prefecture, Changbai Mountain Academy of Sciences, 42°40'51"N, 128°11'48"E, 896 m asl., 6 August 2017, MHHNU 9149 • Jilin Province, Yanbian Korean Autonomous Prefecture, Changbai Mountain, 42°22'61"N, 128°19'42"E, 950 m asl., 7 August 2017, MHHNU 9164.

##### Comments.

In the present phylogenetic analysis, *P.jilinensis* was closely related to accessions of *P.flaccida* and *P.roellinii*. *Phaeoclavulinaroellinii* has an ochraceous to watery ochre-brown fruiting body and *P.flaccida* has a beige fruiting body. Thus, these species cannot be distinguished by color alone, but by other characters. *Phaeoclavulinaroellinii* has long and narrowly clavate basidia (38–70 × 5–7(8) μm), whereas *P.jilinensis* has relatively short basidia (20–36 × 5–7 µm). In addition, *P.roellinii* has abundant stellate crystals adhering to the hyphae, but we have not observed this character in *P.jilinensis*. The color of the fruiting body, sparse branches, and relatively longer basidia (38–45 × 5.5–6.5 vs. 20–36 × 5–7 μm) distinguish *P.flaccida* from *P.jilinensis*.

## ﻿Discussion

In the field, *Phaeoclavulina* species can be easily confused with *Ramaria* or *Clavaria* because in most *Phaeoclavulina* taxa the fruiting body is coralloid or ramaroid. These genera are difficult to distinguish by macroscopic characters alone. However, the following microstructural characters distinguish *Phaeoclavulina* from other coralloid fungi: echinulate or warty spores, strongly cyanophilius; and clamp connections are present. In the past, most species of *Phaeoclavulina* were classified in Ramariasubg.Echinoramaria (Corner 1970; Petersen 1981). Subsequently, *Phaeoclavulina* was transferred to *Gomphus* sensu lato (Giachini et al. 2010). However, in previous studies (Liu et al. 2022; Xu et al. 2022), a monophyletic *Phaeoclavulina* clade is consistently retrieved in phylogenetic analyses and *Phaeoclavulina* is indicated to be phylogenetically separated from the Gomphaceae. Unfortunately, the current molecular evidence does not provide strong support values. To resolve the taxonomic position of *Phaeoclavulina* and relationships among the genera of Gomphaceae, worldwide sampling of taxa and more comprehensive molecular data for phylogenetic analysis are needed.

The present work enriches our knowledge of the genus *Phaeoclavulina* in China. Three new species are described, and the ITS+LSU phylogenetic trees strongly support that each of the species is monophyletic and phylogenetically distinct from other known species of *Phaeoclavulina*. These three new species were collected in different regions of China: *P.bicolor* was collected from a tropical wet climate in Hainan province; *P.echinoflava* was collected in high-altitude area from Tibet; and *P.jilinensis* was collected from a temperate continental climate in Jilin province. Thus, the three areas differ entirely in climate and altitude, indicating that species of *Phaeoclavulina* are widely distributed in China and highly adaptable. Furthermore, the morphological distinctiveness of the three species is unambiguous; for example, *P.echinoflava* has relatively few branches and a bright yellow fruiting body. The shape and color of the *Phaeoclavulina* fruiting body might reflect different climates and altitudes. In addition, records of *Phaeoclavulina* species in China are mainly derived from fungal resource surveys and *Fungi of China* (Teng 1963), many species names may be misused, and there are few or no specimens to verify their authenticity. Thus, we hope that additional samples of *Phaeoclavulina* will be collected in China for construction of a well-supported phylogenetic tree with expanded taxonomic coverage in the future, so as to achieve a more comprehensive understanding of *Phaeoclavulina* and its distribution.

### ﻿Key to known *Phaeoclavulina* species in China

**Table:** 

1	Species pileate	**2**
–	Species coralloid or ramarioid	**3**
2	Hymenium violet	***P.grandis***
–	Pileus green	***P.viridis***
3	Spore spines connected into circular to semi-circular ridges	***P.decolor***
–	Spore spines not connected into circular to semi-circular ridges	**4**
4	Crystals present in trama or adhering to rhizomorphic hyphae	**5**
–	Crystals absent in trama or adhering to rhizomorphic hyphae	**6**
5	Basidiomata bruising green	***P.flaccida***
–	Basidiomata bruising red	***P.eumorpha***
6	Spores verrucose	**7**
–	Spores echinulate	**8**
7	Basidiomata bruising olive or green on handling or weathering	***P.abietina***
–	Basidiomata not bruising olive or green on handling or weathering	***P.sikkimia***
8	Hyphae with H-connection	***P.zippelii***
–	Hyphae without H-connection	**9**
9	Branch tips discolorous with basidiomata	**10**
–	Branch tips concolorous with basidiomata	**11**
10	Spores > 15 µm long	***P.macrospora***
–	Spores < 15 µm long	**12**
11	Basidiomata bright-colored	**13**
–	Basidiomata deep-colored	**14**
12	Branch tips yellowish white	***P.bicolor***
–	Branch tips green or blue	**15**
13	Basidiomata orange or cinnamon	**16**
–	Basidiomata bright-yellow or lemon-yellow	**17**
14	Basidiomata > 11 cm tall	***P.campestris***
–	Basidiomata < 11 cm tall	**18**
15	Tips copper-green	***P.aeruginea***
–	Tips blue	**19**
16	Spores acute spines	***P.cokeri***
–	Spores volcanic spines	***P.cinnamomea***
17	Tips blunt, broom-form or short-digitate	***P.echinoflava***
–	Tips acute, tapered	***P.jilinensis***
18	Hymenium amphigenous	***P.capucina***
–	Hymenium unilateral	**20**
19	Spores < 10 µm long	***P.mutabilis***
–	Spores > 10 µm long	***P.cyanocephala***
20	Spores 4.5–6.5 µm long, basidia 25–36 µm long	***P.curta***
–	Spores 7–11 µm long, basidia 40–50 µm long	***P.longicaulis***

## Supplementary Material

XML Treatment for Phaeoclavulina bicolor

XML Treatment for Phaeoclavulina echinoflava

XML Treatment for Phaeoclavulina jilinensis

## ﻿Additional information

### Author ORCIDs

Peng-Tao Deng https://orcid.org/0000-0002-8755-7965

Wen-Hao Liu https://orcid.org/0000-0002-6937-1446

Zai-Wei Ge https://orcid.org/0000-0003-3184-4604

Ping Zhang https://orcid.org/0000-0002-8751-704X

### Data availability

All of the data that support the findings of this study are available in the main text or Supplementary Information.

## Supplementary materials

Supplementary material 1 Multiple sequence alignment Data type fasta https://binary.pensoft.net/file/1118375This dataset is made available under the Open Database License (http://opendatacommons.org/licenses/odbl/1.0/). The Open Database License (ODbL) is a license agreement intended to allow users to freely share, modify, and use this Dataset while maintaining this same freedom for others, provided that the original source and author(s) are credited.Author: Peng-Tao Deng
